# Azathioprine-Induced Hypersensitivity Syndrome Presenting as Sepsis-Like Syndrome in a Patient With Mucous Membrane Pemphigoid: A Report of a Rare Case

**DOI:** 10.7759/cureus.110461

**Published:** 2026-06-08

**Authors:** Muhammad Usman, Laiba Murtaza, Abdul Basit, Muhammad Zatmar Khan, Muhammad Zawar Asif

**Affiliations:** 1 Internal Medicine, Mercy Hospital Fort Smith, Fort Smith, USA; 2 Internal Medicine, Arkansas College of Osteopathic Medicine, Fort Smith, USA; 3 Internal Medicine, King Edward Medical University, Lahore, PAK; 4 Internal Medicine, Baptist Health System, Fort Smith, USA

**Keywords:** adverse drug reaction, azathioprine therapy, cellcept (mycophenolate mofetil), drug hypersensitivity syndrome, mucous membrane pemphigoid, sepsis-like shock

## Abstract

Azathioprine hypersensitivity syndrome (AHS) is a unique, idiosyncratic drug reaction with typical occurrence within the first 2-4 weeks of azathioprine initiation. It features low-grade fever, gastrointestinal symptoms, malaise, and sometimes life-threatening hemodynamic instability. It commonly masquerades as septic shock with an unknown source of infection or an underlying autoimmune pathology flare-up. We present a case of a woman in her late 60s who was started on azathioprine for a new diagnosis of mucous membrane pemphigoid. The patient presented on two occasions with circulatory shock, lactic acidosis, and acute kidney injury occurring approximately two weeks after azathioprine exposure, including after inadvertent rechallenge. An extensive infectious and endoscopic workup was negative. The diagnosis of AHS was established, given the temporal association of azathioprine exposure and symptom occurrence and a negative alternative etiological workup. The resolution of the patient's symptoms within a few days of drug discontinuation further strengthened the diagnosis. The patient was subsequently transitioned to mycophenolate mofetil for her mucous membrane pemphigoid with no symptom recurrence and appropriate disease control. This case highlights the importance of recognizing AHS as a unique cause of unexplained septic shock-like presentations in patients with recent exposure to azathioprine, and the danger of inadvertent rechallenge of azathioprine. This case also highlights the rare presentation of AHS with significant circulatory collapse, lactic acidosis, and no cutaneous involvement.

## Introduction

Azathioprine is a purine analog and is widely used as an immunosuppressive medication in the management of various autoimmune and inflammatory pathologies, including inflammatory bowel disease, systemic lupus erythematosus, rheumatoid arthritis, organ transplantation, pemphigus vulgaris, and bullous pemphigoid [1]. The mechanism of azathioprine involves inhibition of DNA and RNA synthesis by incorporating toxic metabolites and halting the de novo synthesis pathway [1].

The adverse effects of azathioprine are widely divided into well-characterized dose-dependent side effects (hepatotoxicity and myelosuppression) and less known hypersensitivity-related adverse effects commonly known as azathioprine hypersensitivity syndrome (AHS). AHS is a rare phenomenon occurring in approximately 2% of cases and typically within the first 2-4 weeks of therapy [1]. AHS is characterized by febrile illness, gastrointestinal symptoms including nausea, vomiting, diarrhea, and abdominal pain, generalized fatigue, arthralgias, and cutaneous manifestations such as neutrophilic dermatosis [2,3]. Pancreatitis has also been reported in some cases of AHS [1]. In severe cases, AHS can progress to life-threatening circulatory collapse, multi-organ failure, and anaphylaxis, particularly with rechallenge [3,4].

AHS poses a significant diagnostic complexity because its clinical presentation closely resembles sepsis or an acute flare of underlying autoimmune disease [3]. The fact that AHS is an idiosyncratic reaction that is neither dose-dependent nor related to thiopurine methyltransferase (TPMT) activity further compounds the diagnostic challenge [4].

We present a case of AHS in a patient with mucous membrane pemphigoid who was started on azathioprine and subsequently hospitalized twice with episodes of sepsis-like syndrome requiring vasopressor support in the ICU each time, with the second hospitalization relatively more severe following inadvertent rechallenge of azathioprine. This case is distinguished by the absence of cutaneous manifestations, significant hemodynamic collapse requiring vasopressor support, endoscopic evidence of gastrointestinal manifestations of AHS, and successful transition to mycophenolate mofetil.

## Case presentation

Our patient is a woman in her late 60s with past medical history significant for mucous membrane pemphigoid, essential hypertension, and generalized anxiety disorder who was admitted to the hospital with generalized abdominal pain with nausea, vomiting, and diarrhea ongoing for the past two weeks. The patient presented to the emergency department about four days ago with similar symptoms. Workup at that time was consistent with gastroduodenitis, and the patient was discharged home with supportive management. The patient initially reported symptomatic improvement; however, her symptoms subsequently worsened. Initial vital signs were remarkable for hypotension with blood pressure of 90/68, tachycardia with heart rate of 118 beats per minute, and low-grade fever of 38.1°C (100.6°F). Physical examination was completely unremarkable.

Four weeks before admission, the patient had been diagnosed with mucous membrane pemphigoid and started on azathioprine. Her thiopurine methyltransferase (TPMT) levels were normal prior to initiation of azathioprine.

Initial laboratory work was significant for acute kidney injury, mildly elevated transaminases, and lactic acidosis. No eosinophilia was noted. Laboratory evaluation during hospital stay is summarized in Table 1.

**Table 1:** Laboratory evaluation during the first hospital stay

Test name	Value	Reference range
White blood cells	8.3 K/uL	4.0-10.0 K/uL
Red blood cells	5.47 M/uL	3.93-5.22 M/uL
Hemoglobin	15.7 g/dL	11.2-15.7 g/dL
Hematocrit	48.0%	34.1%-44.9%
Platelets	319 K/uL	182-369 K/uL
Sodium	140 mmol/L	136-145 mmol/L
Potassium	4.5 mmol/L	3.5-5.1 mmol/L
Chloride	104 mmol/L	98-107 mmol/L
Carbon dioxide	17 mmol/L	22-29 mmol/L
Blood urea nitrogen	19 mg/dL	8-23 mg/dL
Creatinine	1.40 mg/dL	0.51-0.95 mg/dL
Glucose	159 mg/dL	74-99 mg/dL
Total protein	6.3 g/dL	6.4-8.3 g/dL
Albumin	3.8 g/dL	4.0-4.9 g/dL
Total bilirubin	1.1 mg/dL	0.0-1.2 mg/dL
Alkaline phosphatase	91 U/L	35-104 U/L
Aspartate aminotransferase	65 U/L	0-32 U/L
Alanine aminotransferase	59 U/L	≤33 U/L
Lipase	20 U/L	13-60 U/L
Erythrocyte sedimentation rate	7 mm/hour	0-29 mm/hour
C-reactive protein	38.4 mg/L	≤5 mg/L
Respiratory pathogen polymerase chain reaction panel	Negative	-
Lactic acid	2.8 mmol/L	≤2.0 mmol/L
Blood culture	No growth at 5 days	-
Urine culture	No growth	-
Stool culture	Negative for enteric pathogens	-
Gastrointestinal pathogen polymerase chain reaction panel	Negative	-
Magnesium	1.4 mg/dL	1.2-2.4 mg/dL
*Helicobacter pylori* stool antigen	Negative	-

A contrast-enhanced CT scan of the abdomen showed mild duodenal thickening along with fluid-filled loops of small bowel suggestive of enteritis, and fatty liver changes were noted.

The patient received initial fluid resuscitation; however, the patient remained hypotensive despite >30 mg/kg. At that point, the patient was started on norepinephrine infusion. Due to concerns of infectious pathology, the patient was started on broad-spectrum antibiotics, which were later discontinued after a negative infectious workup. Azathioprine was not continued during her hospital stay.

With fluid resuscitation and vasopressor support, the patient's hemodynamic instability and lactic acidosis resolved within 48 hours, and on day 3 of hospital admission, the patient was discharged home.

Following discharge, azathioprine remained discontinued for approximately two months while the etiology of her symptoms was being evaluated, and she recovered from her illness. Given the absence of a definitive diagnosis at that time, azathioprine was subsequently restarted by her treating rheumatologist.

Approximately 12 days after resumption of azathioprine, the patient again presented to the hospital with similar complaints of abdominal pain and associated nausea and vomiting, which started suddenly about six hours prior to presentation. The patient's vital signs were significant for hypotension with blood pressure of 92/46, tachycardia with heart rate of 101 beats per minute, and low-grade temperature of 38.2°C (100.8°F). Physical examination this time was remarkable for rebound tenderness to palpation in the epigastric and right lower abdomen quadrant; the remainder of the physical examination was normal.

Initial laboratory workup was significant for acute kidney injury, mildly elevated transaminases, and significant lactic acidosis. No eosinophilia was present again during this hospitalization. Laboratory work during the second hospital stay is summarized in Table 2. 

**Table 2:** Laboratory evaluations during the second hospital stay

Test name	Value	Reference range
Complete blood count		
White blood cells	9.2 K/uL	4.0-10.0 K/uL
Red blood cells	5.65 M/uL	3.93-5.22 M/uL
Hemoglobin	16.0 g/dL	11.2-15.7 g/dL
Hematocrit	48.7%	34.1%-44.9%
Platelets	139 K/uL	182-369 K/uL
Complete metabolic panel		
Sodium	138 mmol/L	136-145 mmol/L
Potassium	4.4 mmol/L	3.5-5.1 mmol/L
Chloride	100 mmol/L	98-107 mmol/L
Carbon dioxide	16 mmol/L	22-29 mmol/L
Blood urea nitrogen	27 mg/dL	8-23 mg/dL
Creatinine	1.40 mg/dL	0.51-0.95 mg/dL
Glucose	164 mg/dL	74-99 mg/dL
Total protein	6.7 g/dL	6.4-8.3 g/dL
Albumin	4.2 g/dL	4.0-4.9 g/dL
Total bilirubin	0.7 mg/dL	0.0-1.2 mg/dL
Alkaline phosphatase	115 U/L	35-104 U/L
Aspartate aminotransferase	63 U/L	0-32 U/L
Alanine aminotransferase	44 U/L	≤33 U/L
Glomerular filtration rate	41 mL/min/1.73 m²	≥60 mL/min/1.73 m²
Urinalysis		
Leukocyte esterase	2+	Negative
Nitrites	Negative	Negative
Urine protein	1+	Negative
Urine glucose	Negative	Negative
Urine ketones	Negative	Negative
White blood cells, urine	11-25/hpf	0-2/hpf
Red blood cells, urine	3-5/hpf	0-2/hpf
Bacteria	1+	Negative
Additional laboratory tests		
Serum lipase	16 U/L	13-60 U/L
C-reactive protein	18.9 mg/L	≤5 mg/L
Respiratory pathogen polymerase chain reaction panel	Pending/negative	-
Lactic acid	8.8 mmol/L	≤2.2 mmol/L
Blood culture	No growth	-
Urine culture	No growth	-
Erythrocyte sedimentation rate	2 mm/hour	0-29 mm/hour
Cytomegalovirus polymerase chain reaction	Negative	-
Cryptococcal antigen (sputum)	Negative	-
Epstein-Barr virus polymerase chain reaction	Negative	-
Gastrointestinal pathogen polymerase chain reaction panel	Negative	-
Magnesium	1.4 mg/dL	1.6-2.4 mg/dL

Contrast-enhanced CT of the abdomen and pelvis showed thickening of the gastric antrum and proximal duodenum, suggestive of gastroduodenitis without any ulceration or perforation.

Due to significant hypotension and lactic acidosis, the patient again received aggressive fluid resuscitation and vasopressor support with norepinephrine, leading to resolution of hemodynamic instability and lactic acidosis within 48 hours.

**Figure 1:**
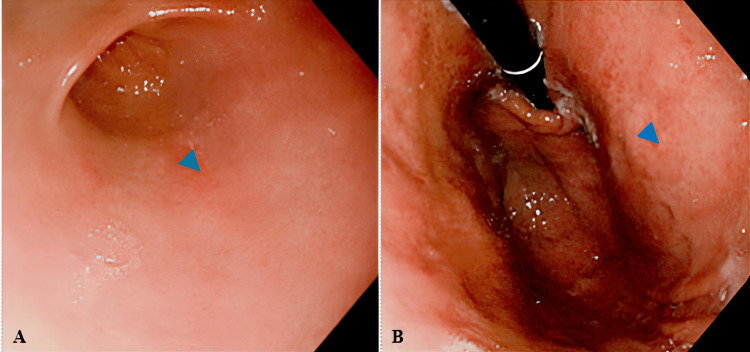
Upper gastrointestinal endoscopy demonstrating erythematous gastritis involving the distal and proximal stomach (A) Endoscopic view of the gastric antrum showing mild mucosal erythema with a patent pyloric channel (blue arrowhead). (B) Endoscopic view of the gastric body showing diffuse mucosal erythema and edema with prominent rugal folds (blue arrowhead).

An esophagogastroduodenoscopy was performed, revealing antral gastritis. A biopsy of the antrum was negative for *H. pylori* and revealed active inflammation in the background of chronic gastritis occurring concurrently with the hypersensitivity episode (Figure 1).

Evaluation of serum tryptase levels (7.2 mcg/L; reference range: <11.0 mcg/L) and human herpesvirus 6 (HHV-6) testing was also unremarkable. Given the negative diagnostic evaluation and the reproducible temporal relationship to azathioprine exposure, a diagnosis of azathioprine-induced hypersensitivity syndrome was made, leading to permanent discontinuation of azathioprine.

The patient was eventually discharged from the hospital with close follow-up with her rheumatologist for her mucous membrane pemphigoid.

At the six-month follow-up, the patient remained asymptomatic while receiving mycophenolate mofetil and had no recurrence of hypersensitivity symptoms.

## Discussion

This case of AHS in the context of azathioprine use for mucous membrane pemphigoid highlights several important clinical aspects.

AHS has been reported most frequently in patients with inflammatory bowel disease and antineutrophil cytoplasmic antibody-associated vasculitis [1]. Reports of AHS in patients with mucous membrane pemphigoid appear to be exceedingly rare, thus highlighting that the risk for AHS is not confined to commonly reported disease populations.

AHS commonly presents with febrile illness, gastrointestinal disturbance, and cutaneous manifestations, including neutrophilic dermatosis. A comprehensive review of 67 cases of AHS found that approximately 49% had cutaneous manifestations, implying that approximately half of the cases, just like ours, presented without any cutaneous manifestations [2,3]. This also highlights that the absence of cutaneous manifestations may have contributed to the delay in the final diagnosis during the first hospitalization in our case.

The diagnosis of AHS was supported by several key facts, including a temporal association between azathioprine initiation and symptom onset (within 3 weeks on first exposure and within 12 days on rechallenge), a positive rechallenge leading to reproduction and rapid escalation of symptoms, and resolution of symptoms upon drug discontinuation on both occasions. Extensive negative workup for alternative etiologies, including infectious, autoimmune, and structural causes, further supported our diagnosis. The normal TPMT levels highlight the idiosyncratic nature of AHS, independent of dose [4].

The hematologic profile in our patient, with marked neutrophilia and no eosinophilia, is consistent with the large cohort AHS study, which reported elevated neutrophils without eosinophilia [5]. This pattern distinguishes AHS from drug reaction with eosinophilia and systemic symptoms (DRESS), which typically features significant eosinophilia. Negative results for HHV-6, cytomegalovirus, and Epstein-Barr virus further differentiate this case from DRESS, in which herpesvirus reactivation is a known defining feature [6]. These findings also suggest that AHS represents a distinct clinical entity from DRESS and is likely mediated by a different immunological mechanism, potentially a type III or type IV hypersensitivity reaction, resulting in neutrophilic activation and T-cell-mediated immune response, respectively [1].

Our case is also unique in terms of the severity of hemodynamic instability. While hypotensive shock has been reported in AHS, it remains a relatively uncommon manifestation, reported in about 8% of cases in one literature review [3]. The degree of hemodynamic compromise in our patient is among the most severe reported in AHS literature. The FDA label for azathioprine does report gastrointestinal hypersensitivity reactions with accompanying hypotension [2,7]. The American College of Chest Physicians guidelines note that azathioprine hypersensitivity reaction can rarely lead to a sepsis-like syndrome [8]. The hemodynamic instability in our case was severe enough to require vasopressor support with norepinephrine on both occasions, meeting criteria for septic shock by clinical definition, despite the absence of infection.

Our case also emphasizes the importance of considering alternative immunosuppressive agents in patients with AHS. Mycophenolate mofetil has been shown to be an effective and well-tolerated alternative to azathioprine for mucous membrane pemphigoid with lower rates of discontinuation secondary to side effects [9]. This approach is supported by our case, in which transitioning to mycophenolate mofetil led to complete resolution of symptoms and no further hospitalizations.

## Conclusions

AHS is an uncommon but potentially life-threatening idiosyncratic reaction to azathioprine that can closely resemble a sepsis-like syndrome, including gastrointestinal disturbance, malaise, febrile illness, arthralgias, and cutaneous manifestations. Clinical presentations without cutaneous manifestations and significant circulatory shock pose significant diagnostic challenges. This case emphasizes that rechallenge with azathioprine after a suspected hypersensitivity reaction should be avoided to prevent more severe and potentially fatal reactions. This case further contributes to the literature on the safety of mycophenolate mofetil in patients with AHS who require permanent discontinuation of azathioprine.
